# Strong Target Attack on Hypergraph Neural Networks via Label Poisoning and Structure Modification

**DOI:** 10.3390/e28030308

**Published:** 2026-03-09

**Authors:** Jie Huang, Qiaoyan Sun, Na Zhang, Meizhu Zheng

**Affiliations:** 1 College of Technology and Data, Yantai Nanshan University, Yantai 265713, China; hj0082@163.com (J.H.); nana101412@163.com (N.Z.); zhengmeizhu8ys@163.com (M.Z.); 2 College of Intelligent Science and Engineering, Yantai Nanshan University, Yantai 265713, China

**Keywords:** hypergraph neural networks, label poisoning, strong target attack, structure modification

## Abstract

Hypergraph Neural Networks (HGNNs) have become an important tool for processing complex structured data due to their ability to model higher-order associative relationships. However, the inherent adversarial vulnerabilities of HGNNs may raise serious security risks. The associated risks are far more pronounced in strong target attacks, which are highly targeted and demand the accurate misclassification of source-class nodes into predefined target classes. Current research on attacks against HGNNs mostly focuses on untargeted attacks or common target attacks, and lacks attacks that precisely control the attack class. Therefore, the research related to strong target attacks is still in an undeveloped state. To fill this research gap, this paper proposes a Strong Target Attack framework for HGNNs based on Label poisoning and Structure modification (STALS). The framework first uses feature similarity and hypergraph structure adaptability to select the optimal target class. Subsequently, the nodes are label-poisoned under the label change budget constraint. A gradient-guided greedy hyperedge reconstruction strategy is used to optimize the association relationship between poisoned nodes and hyperedges within the structure modification budget, maximize the propagation efficiency of mislabeled information, and achieve stable directed misclassification from source class nodes to target classes. We conducted extensive experiments on four mainstream datasets, and the experimental results show that STALS achieves excellent attack performance and significantly outperforms existing baseline methods in terms of success classification rate.

## 1. Introduction

Hypergraph Neural Networks (HGNNs) are advanced learning frameworks that overcome the limitations of traditional Graph Neural Networks (GNNs) in modeling binary relationships [1,2]. By naturally capturing complex multi-entity associations through hyperedge structures, HGNNs demonstrate exceptional representation learning capabilities across diverse domains [3]. Leveraging their efficient modeling of higher-order dependencies, HGNNs have become essential tools for processing complex structured data, finding applications in classification tasks and disease monitoring scenarios [4,5].

However, the inherent adversarial vulnerability of deep learning models also exists in HGNNs [6,7,8]. Attackers can significantly degrade the model’s classification performance by applying minor perturbations to the hypergraph structure, node features, or label information, posing severe security risks [9]. In recent years, researchers have begun addressing adversarial attacks on HGNNs. For instance, HyperAttack, as the first white-box adversarial attack framework targeting HGNNs, leverages gradients and integrated gradients to guide perturbations targeting the hyperedge link states of specific nodes [10]. DGHSA employs gradient-guided modifications to hypergraph structures to achieve untargeted attacks on global model performance [11]. MGHGA leverages momentum gradient mechanisms to alter node features, constructing untargeted poisoning attacks [12]. FMGHA proposes structural attack and hybrid attack variants by integrating future gradients with momentum gradients [13]. While these studies preliminarily reveal HGNNs’ vulnerabilities, untargeted attacks focus on global performance degradation and lack precise control over specific node classification outcomes. Targeted attacks focus on specific node categories, aiming to induce classification errors in specified nodes.

**Figure 1:**
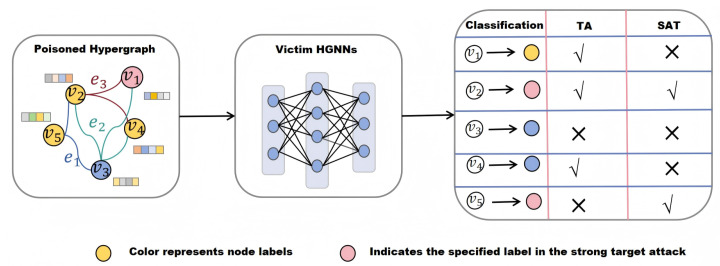
The difference between Target Attack (TA) and Strong Target Attack (STA). Different colors correspond to different labels, and lines connecting multiple nodes represent hyperedges. In the TA, misclassification of a node constitutes a successful attack. In the STA, an attack is successful only if the node is misclassified into the target label.

Compared to traditional target attacks, strong target attacks are a more targeted and highly realistic form of targeted attack. Figure 1 illustrates the difference between target attacks and strong target attacks. In target attacks, attackers only need to perturb these nodes to deviate from their true labels [10]. The attack is deemed successful regardless of the final classification to any other label. For example, if node $v_{2}$ is not classified as the yellow label, the attack succeeds. In strong target attacks, attackers must precisely perturb nodes to strictly classify them into predefined target labels (e.g., pink labels in Figure 1). Nodes $v_{2}$ and $v_{5}$ are classified with pink labels, indicating a successful attack. Strong targeted attacks have clear application scenarios across multiple critical domains, posing more severe potential risks. For example, HGNNs are commonly used in medical diagnostics to analyze gene molecular hypergraphs for early disease screening. If an attacker executes a strong target attack, they can forcefully modify labels of high-risk disease samples (source class) and optimize the association between hyperedges and nodes. This compels HGNNs to misclassify them as healthy samples (target class) instead. Consequently, physicians may misjudge patient health status, miss optimal treatment windows, and directly endanger patient safety.

Despite the significant real-world threat posed by strong target attacks, current research on HGNNs remains largely unexplored. Existing label attack methods for GNNs (such as Lafak [14]) aim to misclassify nodes without controlling the target labels. Furthermore, structural attack approaches fail to consider the synergistic optimization of labels and structure, making precise targeting difficult to achieve.

To address this research gap, we propose a Strong Target Attack for HGNNs based on Label poisoning and Structural modification (STALS), aiming to achieve precise classification control over source label nodes (also known as target nodes). Specifically, we first select the optimal target label through key metrics: feature distribution similarity and hypergraph structural adaptability. Subsequently, source label nodes undergo label poisoning under the constraints of the label modification budget. Through a gradient-guided greedy hyperedge reconstruction strategy, the binary association between poisoned nodes and hyperedges is iteratively optimized within the structural modification budget to maximize the propagation efficiency of erroneous label information. Additionally, we designed the variant model STALS-Deg, which further enhances attack efficiency by prioritizing the poisoning of nodes with high nodal degrees.

The main innovations of this paper are as follows:

- We propose the first Strong Target Label Attack framework for HGNNs (STALS), overcoming the limitation of existing attacks that cannot precisely control target classes. STALS enables directed misclassification of source label nodes to specified target labels.
- We design a multi-dimensional optimal target label selection mechanism that integrates feature similarity and hypergraph structural adaptability metrics.
- We establish a joint optimization paradigm for label poisoning and structural reconstruction.
- We compare STALS and its variants with several state-of-the-art attack methods across multiple datasets and HGNNs, demonstrating the superiority of our approaches.

This paper is organized as follows: Section 2 reviews the model evolution and application scenarios of HGNNs, along with the current state of adversarial attacks on image, text, and graph data. Section 3 introduces the concepts of hypergraphs and HGNNs, while elaborating on the threat model of strong adversarial attacks. Section 4 introduces the STALS framework, including its time complexity and algorithm. Section 5 validates the proposed method’s attack performance and transferability through comparative experiments. Finally, Section 6 summarizes the research findings and identifies future extension directions.

## 2. Related Work

### 2.1. Related Work on Hypergraph Neural Networks

In recent years, research on HGNNs has centered on model architecture optimization, scalability enhancement, and multi-scenario applications, forming a multidimensional technical framework.

Regarding model architecture design, early HGNNs achieved feature aggregation at the hyperedge level through normalized hypergraph Laplacian operators, constructing multi-layer hyperedge convolutional structures to capture local and global high-order correlations [15]. Subsequent research further expanded the representational capabilities of HGNNs [16]. Some approaches introduced attention mechanisms (e.g., Hypergraph Attention [17]), dynamically assigning association weights between hyperedges and nodes to enhance the precision of feature aggregation. Other studies combined contrastive learning strategies (e.g., CHGNN [18]), designing adaptive hypergraph view generators and hyperedge homogeneity-aware encoders to effectively exploit unlabeled data for optimizing node embeddings. To address specific task requirements, HGNNs also spawned multimodal fusion variants (e.g., MHNet [19]), employing parameterized polynomial filtering and feature sampling strategies to process multi-source heterogeneous data. In hyperspectral image classification, EHGNN addresses limitations in semantic information representation and pixel-level spectral-spatial information retention by introducing key hypergraph concepts and an end-to-end CNN-HGNN fusion architecture [4]. FastHGNN proposes a hierarchical node-hyperedge sampling strategy, reformulating hypergraph convolutions into double integral forms and achieving discretization approximations [20]. The PCL method combines hypergraph partitioning with contrastive learning to enable efficient training of hypergraphs with tens of millions of nodes, outperforming full-hypergraph training results. Moreover, the t-HGSP framework learns hypergraph topology via tensor representation learning [1]. By minimizing total variation (TVL-HGSP) and optimizing hypergraph connectivity (PDL-HGSP), it adaptively learns robust hypergraph structures from data, offering solutions for scenarios lacking explicit hypergraph priors [3].

### 2.2. Related Work on Adversarial Attacks

Adversarial attacks aim to mislead deep learning models into making erroneous decisions by applying minute perturbations to data [21]. Research in this field has expanded across diverse data types including images, text, and graph, with increasingly in-depth exploration into hypergraphs [22,23,24]. This section first reviews attack studies on other data types, followed by an introduction to adversarial attacks based on graph data.

Research on adversarial attacks against image data began early and has matured technologically [6,25]. The challenge lies in ensuring the transferability of attacks across different models while maintaining the visual invisibility of perturbations. FGSM calculates the gradient sign of the loss function for input images and applies single-step perturbations to generate adversarial examples. While efficient, its attack strength is limited [26]. PGD enhances attack effectiveness and stability through multi-step iterative gradient updates projected within perturbation budget constraints, establishing itself as the benchmark method for image adversarial attacks [27]. ALDA uses Grad-CAM to identify model attention regions, optimizing the perturbation process through an attention-guided look-ahead mechanism combined with attention-disturbing data augmentation strategies [22]. NGI-Attack extracts gradient information from the neighborhood surrounding clean images [28].

The discrete nature and semantic sensitivity of text data pose unique challenges for adversarial attacks [29,30]. Modifications must alter only a small number of text tokens while preserving semantic integrity and syntactic correctness. Word-level attacks are currently mainstream. AdaptiveWordBug proposes an Adaptive Scoring Strategy that automatically adjusts method weights to precisely identify keywords in diverse texts, outperforming baseline methods [23]. TextJosher employs a transfer-based black-box attack framework, utilizing local surrogate models to estimate gradients and compute embedding-level saliency to identify key tokens [29].

Traditional graph data adversarial attacks (where edges connect only two nodes) focus on structural perturbations (adding/removing edges) and node feature modifications, requiring preservation of graph topological properties (such as node degree and connectivity) [31,32,33]. Untargeted attacks aim to degrade the model’s overall performance [34]. MetaAttack employs meta-learning to optimize attack strategies, rapidly identifying optimal perturbation combinations within limited budgets [35]. Targeted attacks focus on specific nodes. Nettack combines gradient descent with graph topology constraints, achieving directed misclassification by modifying the neighbor structure and features of target nodes [36]. GraphZOOM targets inductive GNNs, employing zero-order optimization to solve node selection and feature perturbation problems [37]. NAG-R incorporates a Nesterov accelerated gradient to avoid local optima, combining reconnection operations to preserve fundamental graph properties [38]. This approach enhances attack success rates and transferability while ensuring perturbation concealment. NEAttack adopts an information entropy perspective [39]. By quantifying the feature smoothness of node-related subgraphs, it constructs an optimization objective that integrates node entropy loss and CW loss.

## 3. Preliminary

For the reader’s convenience, Table 1 summarizes the parameters frequently used in this paper.

### 3.1. Hypergraph

Hypergraphs are higher-order topological structures that transcend the limitations of traditional graphs (where edges connect only two nodes). They naturally model the associative relationships among multiple entities in real-world scenarios and serve as the foundational topological structure for HGNNs. Let a hypergraph be defined as a tuple H=(V,E,X), where $V=\{v_{1},v_{2},\ldots ,v_{N}\}$ is the node set, with N=|V| denoting the total number of nodes. $E=\{e_{1},e_{2},\ldots ,e_{M}\}$ denotes the set of hyperedges, where M=|E| represents the total number of hyperedges. $X\in R^{n\times d}$ denotes the node feature matrix, where *d* is the feature dimension. In a hypergraph, the degree $d(v_{i})$ of a node represents the number of hyperedges containing that node, i.e., $d\left(v_{i}\right)=\left|\left\{e_{j}\in E|v_{i}\in e_{j}\right\}\right|$. The node degree diagonal matrix can be represented as $D_{v}\in R^{N\times N}$, where $D_{v[i,i]}=d\left(v_{i}\right)$. The adjacency matrix of a hypergraph, denoted as $H\in R^{N\times M}$, clearly characterizes the membership relationship between nodes and hyperedges. If node $v_{i}$ belongs to hyperedge $e_{j}$, then $H_{i,j}=1$. Otherwise, $H_{i,j}=0$.

### 3.2. Hypergraph Neural Networks

HGNNs aim to leverage higher-order associations among hyperedges to achieve effective feature aggregation and updating for nodes. HGNNs achieve balanced hyperedge aggregation through the normalization of the hypergraph Laplacian operator, expressed as:

(1) $$\tilde{\Delta }=D_{v}^{-1/2}HWD_{e}^{-1}H^{T}D_{v}^{-1/2}.$$

where $W\in R^{M\times M}$ is the hyperedge weight matrix, $D_{e}=diag\left(\delta \left(e_{1}\right),...,\delta \left(e_{M}\right)\right)R^{M\times M}$ is the hyperedge degree matrix. Hyperedge convolution is the operation in HGNNs for extracting higher-order correlation features. It achieves feature aggregation by normalizing the hypergraph Laplacian operator, expressed as:

(2) $$HConv\left(X_{in},\Theta \right)=\tilde{\Delta }X_{in}\Theta .$$

where $X_{in}\in R^{n\times h_{in}}$ is the input feature matrix, and $\Theta \in R^{h_{in}\times h_{out}}$ is the convolution parameter. $HConv\left(X_{in},\Theta \right)\in R^{N\times h_{out}}$ is the feature matrix after convolution, incorporating hyper-edge correlation information.

HGNNs progressively abstract local and global correlation features by stacking *L* layers of super-edge convolution modules, constructing an end-to-end feature aggregation process. For layer *l*, the feature update logic is as follows:

(3) $$X^{\left(l\right)}=\mathrm{ReLU}\left(\Delta X^{\left(l-1\right)}\Theta^{\left(l\right)}\right).$$

where $X^{\left(l\right)}\in R^{N\times h_{l}}$ denotes the embedding matrix of the l-th layer’s output, $h_{l}$ represents the feature dimension of the *l*-th layer. ReLU(·) is the nonlinear activation function employed to introduce the learning capability for complex nonlinear relationships.

To embed the final node into a mapping representing a probability distribution over categories, HGNNs employ the Softmax function to achieve classification mapping, expressed as:

(4) $$Z=\mathrm{Softmax}(X^{\left(L\right)}W_{cls}+b_{cls}).$$

where $X^{\left(L\right)}$ denotes the final node embedding matrix. $W_{cls}\in R^{h_{L}\times C}$ represents the classification weight matrix, and $b_{cls}\in R^{C}$ denotes the classification bias vector, with *C* being the total number of classes. $Z\in R^{N\times C}$ is the label probability matrix, where $Z_{i,c}$ denotes the probability that node $v_{i}$ belongs to category *c*, satisfying $\sum_{c=1}^{C}Z_{i,c}=1$. Its form is:

(5) $$Z_{i,c}=\frac{\exp(X_{i}^{\left(L\right)}W_{cls}\left[:,c\right]+b_{cls}\left[c\right])}{\sum_{k=1}^{C}\exp\left(X_{i}^{\left(L\right)}W_{cls}\left[:,k\right]+b_{cls}\left[k\right]\right)}.$$

HGNNs are typically applied to semi-supervised node classification tasks, using label information from labeled nodes to optimize model parameters. They employ a cross-entropy loss function to measure the discrepancy between predicted probabilities and true labels, expressed as:

(6) $$L=-\frac{1}{|V_{train}|}\sum_{v_{i}\in V_{train}}\sum_{c=1}^{C}I\left(y_{i}=c\right)\log\left(Z_{i,c}\right).$$

where $V_{train}$ denotes the set of labeled nodes, $|V_{train}|$ denotes the number of labeled nodes. $y_{i}$ represents the true label of node $v_{i}$. I(·) is the indicator function, where $I(y_{i}=c)=1$ if $y_{i}=c$ and 0 otherwise. The loss function minimizes classification errors on labeled nodes, guiding the model to learn the associative patterns between hypergraph structures and category labels.

### 3.3. Threat Model

In traditional target attacks, the attacker aims to cause misclassification of the target node. Strong target attacks differ by specifying the source class node and then forcing it to be classified into a fixed target class $y^{*}$. In other words, strong target attacks achieve precise control over the attack category.

In this paper, we propose STALS, a strong target-based attack for HGNNs. STALS maximizes the classification accuracy of misclassifying target nodes into target labels by modifying node labels and structures during training. Formally, the mathematical expression can be represented as:

(7) $$\begin{array}{cc} & Max\sum_{v\in V_{y_{t}}}I\left({\hat{y}}_{v}=y^{*}\right),\\ & s.t.\;{\hat{y}}_{\nu }=f_{\theta^{*}}\left(H^{\prime },X,Y^{\prime }\right),\theta^{*}=\arg\min L\left(\theta ;H^{\prime },X,Y^{\prime }\right),|Y^{\prime }-Y\left|\leq \Delta_{L},\right|H^{\prime }-H|\leq \Delta_{H}. \end{array}$$

where $y^{*}$ denotes the attacker’s pre-specified labels, where ${\hat{y}}_{v}$ represents the set of labels for node $y_{v}$. $\hat{y}$ serves as the predicted label, $\Delta_{L}$ as the label modification budget to control the number of node labels altered.

In addition, to clearly illustrate the distinction between targeted attacks and strong targeted attacks, Equation (8) presents the mathematical expression for targeted attacks.

(8) $$\begin{array}{cc} & Max\sum_{v\in V_{y_{t}}}I\left({\hat{y}}_{\nu }\neq y_{v}\right),\\ & s.t.\;{\hat{y}}_{\nu }=f_{\theta^{*}}\left(H^{\prime },X,Y^{\prime }\right),\theta^{*}=\arg\min L\left(\theta ;H^{\prime },X,Y^{\prime }\right),|Y^{\prime }-Y\left|\leq \Delta_{L},\right|H^{\prime }-H|\leq \Delta_{H}. \end{array}$$

where $y_{v}$ denotes the true label of node *v*. Comparing Equations (7) and (8) reveals that strong target attacks cause nodes to be misclassified into a fixed target class rather than any arbitrary incorrect class. In target attacks, attackers cannot predict the specific misclassified category in advance; they can only ensure the node is misclassified, lacking precise control over the attack’s consequences.

## 4. Methodology

**Figure 2:**
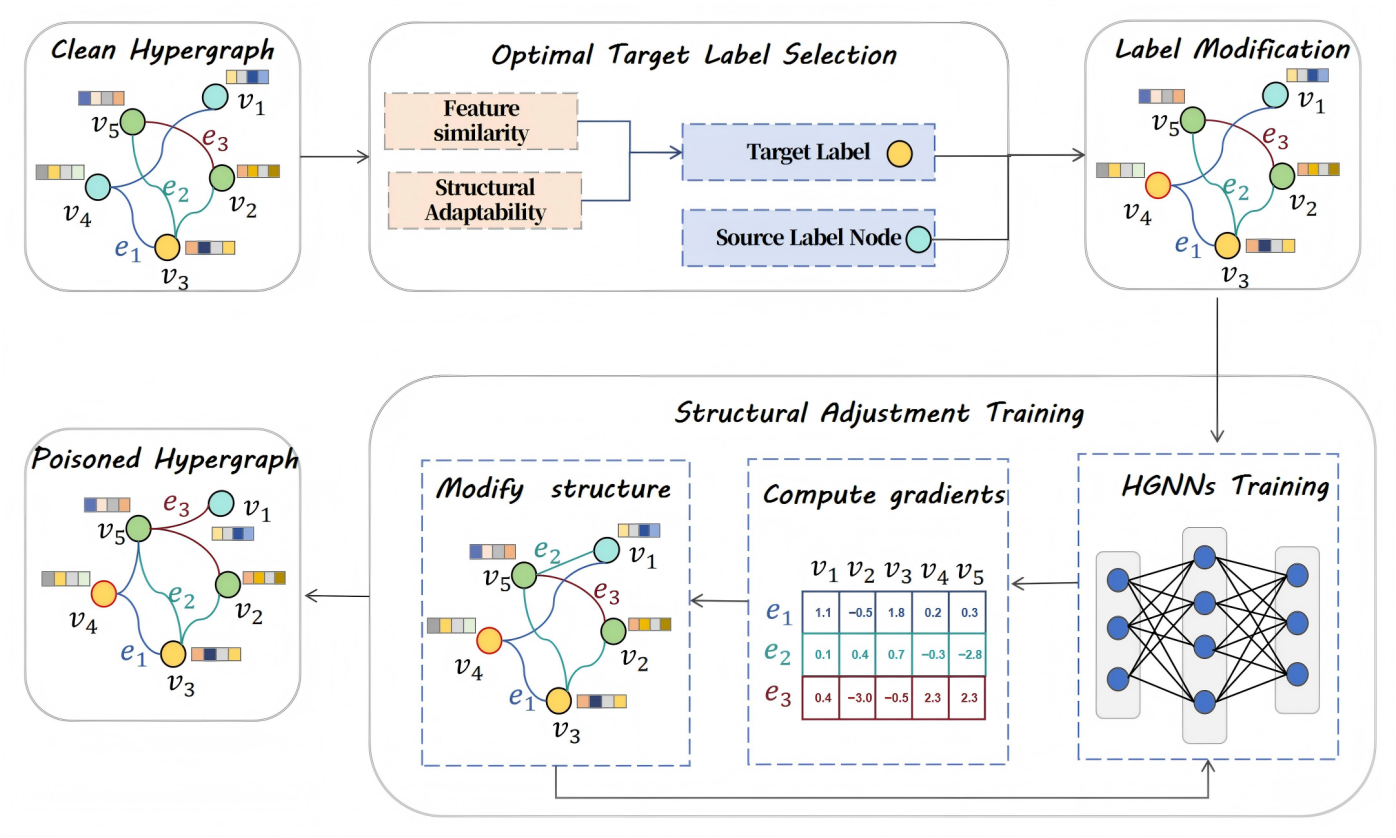
Illustration of our proposed STALS framework. First, STALS selects target labels using clean graph node feature similarity and structural matching metrics. It then embeds the target labels into the source label nodes. Subsequently, HGNNs are trained to compute gradients of the association matrix. Structural modifications are made through gradient-modified rules. Iterative modifications continue until the budget is satisfied, yielding the poisoned hypergraph.

We propose a strong target attack model for HGNNs based on label poisoning and structural modification, named STALS. It aims to achieve efficient strong target attacks on HGNNs through precise target label selection and hypergraph structure optimization. The STALS framework is illustrated in Figure 2. Specifically, the STALS model comprises two core modules: the Optimal Target Label Selection Module and the Structural Adjustment Training Module. The former quantifies the correlation between node features and hypergraph structure to identify the most vulnerable target labels. The latter leverages gradient information to fine-tune node-hyperedge relationships based on label modifications, amplifying attack effectiveness and ensuring stable classification of target nodes into target labels.

### 4.1. Optimal Target Label Selection

Strong target attacks ensure that source label nodes are misclassified into specific target labels rather than arbitrary incorrect labels. Traditional label attack methods fail to account for varying attack difficulties across target labels, randomly or blindly selecting target labels, resulting in inefficient attacks. In reality, target label selection is influenced by multiple factors, for example, the similarity of feature distributions between source and target labels. The closer the feature distributions, the easier it is for the model to confuse the two types of nodes, resulting in lower attack costs. Additionally, the hypergraph shows structural characteristics of the target label.Topological properties such as hypergraph density and node connection strength determine the propagation efficiency of mislabeled information.

Therefore, STALS constructs a comprehensive attack difficulty scoring system by quantifying these key factors to select the optimal target label. Specifically, we first extract feature embeddings from all training nodes to build statistical and structural features for each label.

Let the attacker-specified source label be $y_{source}$, with statistical characteristics $\left(\mu_{s},\rho_{s}\right)$ (where *s* denotes $y_{source}$). Based on the above statistics, for each candidate target label $t\neq y_{source}$, we construct a comprehensive attack difficulty score S(t) by integrating factors such as feature similarity, structural adaptability, and sample size. A lower score indicates easier attack difficulty. The specific calculation is as follows:

Feature similarity factor: Combines the Euclidean distance and cosine similarity to comprehensively measure the similarity between source and target labels. The expression is:

(9) $$Sim\left(s,t\right)=\frac{2\cdot (1+\cos\left(\mu_{s},\mu_{t}\right))}{1+∥\mu_{s}-\mu_{t}{∥}_{2}}.$$

Here, $\cos\left(\mu_{s},\mu_{t}\right)=\frac{\mu_{s}^{⊤}\mu_{t}}{{∥\mu_{s}∥}_{2}\cdot {∥\mu_{t}∥}_{2}}$ represents the cosine similarity, where ${∥\cdot ∥}_{2}$ denotes the L2 norm. Sim(s,t)∈(0,1]. A higher value indicates greater feature similarity and lower attack difficulty. $\mu_{c}$ denotes the label feature mean, which characterizes the central distribution of label features, reflecting the overall feature trend of the label. Its expression is:

(10) $$\mu_{c}=\frac{1}{|V_{c}|}\sum_{v_{i}\in V_{c}}F_{i},\;s.t.\;c\in \left\{s,t\right\}.$$

where $F_{i}\in R^{h}$ is the feature embedding vector of node $v_{i}$, and *h* is the embedding dimension.

Structural adaptation factor: Quantifies the difference in hypergraph density between the source and target labels. A smaller density difference facilitates easier propagation of mislabeled information between nodes of the two labels. The expression is:

(11) $$Str\left(s,t\right)=\frac{1}{1+|\rho_{s}-\rho_{t}|}.$$

where Str(s,t)∈(0,1]. A higher value of Str(s,t) indicates better structural adaptability and lower attack difficulty.

$\rho_{c}$ denotes hypergraph density. It describes the degree of connectivity among nodes of label c within the hypergraph. Higher density indicates faster and broader propagation of label information among nodes within that label. The expression is:

(12) $$\rho_{c}=\frac{\sum_{e_{j}\in E}{\left|e_{j}\cap V_{c}\right|}^{2}}{|V_{c}\left|\cdot \right|E_{c}|},s.t.\;c\in \left\{s,t\right\}.$$

Among these, $E_{c}=\left\{e_{j}\in E|e_{j}\cap V_{c}\neq \emptyset \right\}$ denotes the set of hyperedges containing at least one node from $V_{c}$. The numerator quantifies the association strength of nodes in $V_{c}$ within the hyperedge, while the denominator normalizes by node count and hyperedge count to mitigate statistical bias from scale differences.

Comprehensive scoring function: By integrating the aforementioned factors with the sample size factor, we construct an attack difficulty score:

(13) $$S\left(t\right)=\frac{1}{Sim(s,t)\cdot Str(s,t)}.$$

This score comprehensively reflects the impact of feature similarity and structural adaptability on attack difficulty. A lower score indicates that target label *t* is more suitable as an attack target. We then select the label with the lowest composite score as the optimal target label $y^{*}$, defined as:

(14) $$y^{*}=\arg\min_{t\in \left\{0,...,C-1\right\},t\neq y_{source}}S\left(t\right).$$

After obtaining the target labels, we modify the node labels to generate the poisoned label set. Based on the label modification budget α∈[0,1] (which controls the proportion of nodes undergoing label modification), we select the set of nodes to be poisoned $V_{poison}$ from the source label training nodes $V_{s}=\left\{v_{i}|y_{i}=y_{source},train\_mask\left[i\right]=1\right\}$. The number of poisoned nodes is calculated as: $|V_{poison}|=\max\left(1,\lfloor \alpha \cdot |V_{s}|\rfloor \right)$ where ⌊·⌋ denotes the floor function, ensuring at least one node is poisoned to initiate the attack. Label flipping is performed on nodes in $V_{poison}$, modifying their original labels $y_{source}$ to the optimal target labels $y^{*}$, yielding the poisoned training labels $y_{train}^{\prime }\in {\left\{0,...,C-1\right\}}^{|V_{train}|}$.

### 4.2. Structural Adjustment Training

Attacks relying solely on label modification have inherent limitations: nodes sharing the same hyperedge in a hypergraph typically exhibit similar features and label attributes. When labels are altered, the consistency between the poisoned node and its original hyperedge is disrupted, making it difficult for mislabeled information to propagate effectively.

To address these issues, STALS employs a gradient-guided greedy hyperedge reconstruction strategy. First, it injects misinformation through label poisoning and calculates the attack loss for HGNNs. Then, it differentiates the association between poisoned nodes and hyperedges, using gradient information to identify hyperedge adjustment directions that minimize loss. Under structural modification budget constraints, a greedy strategy sequentially selects the association pairs with the largest absolute gradient values for binary adjustments until the budget is exhausted. Finally, iterative optimization achieves a sustained reduction in attack loss, ensuring stable classification of source label nodes into target labels.

The hyperedge reconstruction lies in maximizing loss reduction at each step by sequentially selecting the association pairs with the largest absolute gradient values through a greedy strategy, subject to the structural modification budget $\Delta_{S}$ (the maximum number of adjustable association pairs). The specific implementation is as follows:

The attack loss for HGNNs is computed and solved for the gradient of the loss with respect to the poisoned node-hyperedge association.

The attack loss $L_{attack}$ employs the cross-entropy loss, aiming to minimize the classification loss corresponding to poisoned labels. This forces the model to learn erroneous associations, expressed as:

(15) $$L_{attack}=-\frac{1}{|V_{train}|}\sum_{v_{i}\in V_{train}}I\left({y_{train}}_{i}^{\prime }=y^{*}\right)\log\left(Z_{i,y^{*}}\right).$$

The loss directly reflects the probability of the poisoned node being classified as $y^{*}$. A smaller loss indicates a more effective attack. Gradient computation is achieved via the chain rule, with the specific expression being:

(16) $$\nabla_{H[i,j]}L_{attack}=\frac{\partial L_{attack}}{\partial Z}\cdot \frac{\partial Z}{\partial X^{\left(L\right)}}\cdot \frac{\partial X^{\left(L\right)}}{\partial \tilde{\Delta }}\cdot \frac{\partial \tilde{\Delta }}{\partial H}{|}_{\left[i,j\right]}.$$

The larger the absolute value of the gradient, the more significant the contribution of this edge correlation adjustment to loss reduction.

If $\nabla_{H[i,j]}L_{attack}<0$: Increasing the association between $v_{i}$ and $e_{j}$ (changing H[i,j] from 0 to 1) reduces the loss, benefiting the attack. If $\nabla_{H[i,j]}L_{attack}>0$: Reducing the association between $v_{i}$ and $e_{j}$ (changing H[i,j] from 1 to 0) reduces the loss, benefiting the attack. For the current hypergraph $H_{curr}$, the gradient is recalculated $\nabla_{H_{curr}\left[i,j\right]}L_{attack}$ for all poisoned nodes $v_{i}\in V_{poison}$ and hyperedges $e_{j}\in E$.

The association pair $\left(i^{*},j^{*}\right)$ is selected with the largest absolute gradient value in *H*, that is:

(17) $$\left(i^{*},j^{*}\right)=\arg\max_{(i,j)\in H}\left|\nabla_{H_{curr}\left[i,j\right]}L_{attack}\right|$$

The association status is adjusted based on the gradient sign:

If $\nabla_{H_{curr}\left[i^{*},j^{*}\right]}L_{attack}<0$: Set $H_{curr}\left[i^{*},j^{*}\right]=1$ (add association);

If $\nabla_{H_{curr}\left[i^{*},j^{*}\right]}L_{attack}>0$: Set $H_{curr}\left[i^{*},j^{*}\right]=0$ (remove association).

### 4.3. Overall Optimization Objective

The ultimate optimization objective of STALS is to minimize the attack loss $L_{attack}$ and maximize the proportion of source label nodes classified into $y^{*}$ within the test set under the constraints of the label change budget and structural modification budget. The mathematical expression is:

(18) $$\begin{array}{l} \min_{H^{\prime }}L_{attack}\;s.t.\;\max_{\beta ,f}\frac{1}{|V_{s}^{test}|}\sum_{v_{i}\in V_{s}^{test}}I\left(Z_{i,y^{*}}=\max_{c\in \{0,...,C-1\}}Z_{i,c}\right)\\ |Y^{\prime }-Y\left|\leq \Delta_{L},\right|H^{\prime }-H|\leq \Delta_{H}. \end{array}$$

where $H^{\prime }$ denotes the poisoned hypergraph.

### 4.4. Algorithm and Time Complexity

Algorithm 1 illustrates the framework of STALS.
**Algorithm 1** STALS: Strong Target Attack via Label Poisoning and Structure Modification**Require:** Hypergraph G=(V,E,X); Source class $c_{source}$; Budget factors α,β; Iterations *T*; Surrogate HGNNs $f_{\theta }$**Ensure:** Poisoned hypergraph $G^{\prime }=\left(V,E^{\prime },X\right)$; Poisoned labels $Y^{\prime }$  1:Initialize t←0, $E^{\prime }\leftarrow E$, $Y^{\prime }\leftarrow Y$, adjusted_count←0  2:$V_{s}\leftarrow \left\{v_{i}|y_{i}=c_{source},train\_mask\left[i\right]=1\right\}$, $|V_{poison}\left|\leftarrow \max(1,\lfloor \alpha \cdot \right|V_{s}\left|\rfloor \right)$  3:$\Delta_{S}\leftarrow \beta \cdot \left|E\right|$ (structural modification budget)  4:**while** t<T and $adjusted\_count<\Delta_{S}$ **do**  5:    Compute $\mu_{s},\rho_{s}$ (feature mean and hypergraph density of $V_{s}$)  6:    Initialize min_score←+∞, $c_{target}\leftarrow 0$  7:    **for** each $t_{cand}\neq c_{source}$ **do**  8:        Compute $\mu_{t},\rho_{t}$ (feature mean and density of $t_{cand}$)  9:        Calculate $Sim\left(s,t\right)=\frac{2(1+\cos\left(\mu_{s},\mu_{t}\right))}{1+∥\mu_{s}-\mu_{t}{∥}_{2}}$, $Str\left(s,t\right)=\frac{1}{1+|\rho_{s}-\rho_{t}|}$10:        $S\left(t\right)=\frac{1}{Sim(s,t)\cdot Str(s,t)}$, update $c_{target}$ if S(t)<min_score11:    **end for**12:    $V_{poison}\leftarrow RandomSample(V_{s},|V_{poison}\left|\right)$, flip labels of $V_{poison}$ to $c_{target}$13:    Train $f_{\theta }$ on $\left(E^{\prime },X,Y^{\prime }\right)$, compute $Z=Softmax(f_{\theta }^{embed}\left(E^{\prime },X\right)W_{cls}+b_{cls})$14:    Calculate $L_{attack}$ and gradient $\nabla_{H[i,j]}L_{attack}$15:    Select $\left(i^{*},j^{*}\right)=\arg\max_{v_{i}\in V_{poison},e_{j}\in E^{\prime }}\left|\nabla_{H[i,j]}L_{attack}\right|$16:    Adjust $E^{\prime }\left[i^{*},j^{*}\right]$ (1 if gradient < 0, else 0)17:    adjusted_count←adjusted_count+1, t←t+118:**end while**

The time complexity of STALS is determined by the two core modules (optimal target label selection and structural adjustment training) and the iterative optimization process. (1) Optimal Target Label Selection Module. Calculating the feature mean of each class involves traversing all node feature vectors, with a time complexity of O(Nd). Computing the hypergraph density of each class requires traversing all hyperedges to count node-hyperedge intersections, with a time complexity of O(CM). The label poisoning operation involves only random sampling and label flipping, with negligible complexity. (2) Structural Adjustment Training Module. Each iteration involves 2-layer hyperedge convolutions and gradient backpropagation via the chain rule. The time complexity per iteration is $O(NM\bar{h})$ ($\bar{h}$ is the average feature dimension across network layers). The greedy selection of optimal node-hyperedge pairs involves traversing βNM pairs, leading to a time complexity of O(βNM) per iteration.

Overall Time Complexity: Considering *T* attack iterations, the total time complexity of STALS is: $O(TNM\left(\bar{h}+\beta \right)+Nd+CM)$.

## 5. Experiments

### 5.1. Datasets Statistics

To comprehensively evaluate the attack’s performance, we conducted integrated experiments on four common graph datasets: Cora [40], Cora-ML [41], Citeseer [42], and Pubmed [43]. These datasets span a wide range of graph sizes, encompassing both small and large structures and exhibit varying levels of network density and feature dimensionality. This ensures our evaluation reflects diverse real-world application scenarios. Detailed statistics for the datasets are presented in Table 2. The node-hyperedge associations were generated by HGNNs.

### 5.2. Baselines

We propose a strong adversarial attack that remains unexplored in existing HGNNs research. Therefore, we made every effort to identify relevant baseline models. The baseline models are detailed as follows.

Random: Random is a simple yet effective attack method that randomly modifies node labels and structures.

Deg: In hypergraphs, node degree is a crucial topological property often used to measure node importance. Deg modifies the labels and structures of nodes with higher degrees.

Nettack [36]: A classic targeted attack method for GNNs that achieves directed misclassification by combining gradient descent with graph topological constraints.

DGHSA [11]: An untargeted attack framework for HGNNs that adopts a derivative graph-based gradient-guided hypergraph structure modification strategy. It degrades the model’s global classification performance by perturbing the hypergraph topology.

Lafak [14]: Lafak is the first label attack algorithm. It overcomes the inherent challenges of dual-level optimization by transforming non-differentiable objectives to achieve attacks.

STALS-Deg: STALS-Deg is a variant of STALS. Unlike STALS, it modifies labels specifically among nodes with high degrees.

### 5.3. HGNN Models

We primarily use the HGNN-KNN to validate the STALS’s effectiveness. Additionally, we use HGNN-ε to demonstrate the transferability of attacks. Both HGNNs are introduced as follows.

HGNN-KNN [44]: HGNN-KNN is a hypergraph neural network integrated with the K-Nearest Neighbors (KNN) algorithm. During hypergraph construction, the KNN algorithm determines node connections for each node; it selects the K most similar nodes in the feature space to form hyperedges.

HGNN-ε [44]: HGNN-ε is a hypergraph neural network that connects hyperedges when node feature similarity falls within the range ε. HGNN-ε constructs hyperedges based on node feature similarity. When the similarity between two or more nodes is within the ε range, a hyperedge connects them.

### 5.4. Parameters and Metrics

For the specific parameter settings of HGNNs: The K parameter in HGNN-KNN is set to 10, and the ε parameter in HGNN-ε is set to 0.5. The number of layers in HGNNs is set to 2, the learning rate is 1 ×$10^{-4}$, the decay factor is 5 ×$10^{-4}$, and the feature dimension is 128. For the specific configuration of STALS parameters: the target labels are as shown in Table 2. The label attack budget is set to $\Delta_{L}=\alpha \left|V_{S}\right|$, where α is set to 0.1; $V_{S}$ is the set of source label nodes. The structural attack budget is set to $\Delta_{S}=\beta M$, where β is set to 0.05.

The objective of STALS is to focus on the directed misclassification effect of unmanipulated clean source-class nodes and verify the generalization ability of the attack. We use the Success Classification Rate (SCR) to quantify the effectiveness of attack methods, where the calculation range is strictly limited to unpoisoned clean source-class nodes (excluding poisoned nodes). A higher value of this metric indicates that the attack can more effectively induce the model to misclassify clean source-class nodes into the target label, reflecting better attack generalization and performance.

(19) $$SCR=\frac{\sum_{v\in V_{S}^{clean}}I\left({\hat{y}}_{v}=y^{*}\right)}{\left|V_{S}^{clean}\right|}.$$

where $V_{S}^{\mathrm{clean}}$ denotes the set of unpoisoned clean source-class nodes, $y^{v}$ is the predicted label of node *v* by HGNNs after the attack, and $y^{*}$ is the predefined target label.

### 5.5. Main Experiments

In this section, we investigate performance under each source label, with results shown in Table 3. The results demonstrate that our proposed models achieve better performance compared to other benchmark models.

For instance, on Citeseer, when the source label is set to 2, the classification accuracies for Deg, Nettack, DGHSA, Lafak, and STALS are 38.12%, 46.52%, 41.96%, 50.30%, and 89.75%, respectively. Similar results are observed across other datasets. STALS achieves this by calculating the easiest label to classify each time and then performing a label attack. During training, the erroneous label information rapidly propagates through hyperedges to numerous connected nodes, affecting feature updates and representation learning on these nodes. This causes bias in the node representations across the entire hypergraph, leading the model to classify victim nodes as the target label. Consequently, Lafak’s performance is lower than STALS.

Additionally, different node selection methods impact attack performance. In the Cora-ML dataset, STALS-Deg achieves a classification accuracy 1.99% higher than the average STALS performance. In HGNNs, high-degree nodes connect multiple hyperedges and occupy a central position in information propagation and aggregation. Label information propagates through hyperedges to numerous connected nodes. When attacking high-degree nodes, their erroneous labels rapidly spread via hyperedges to a large number of neighboring nodes. This disrupts feature updates and representation learning, causing significant bias in the entire hypergraph’s node representations. Consequently, STALS-Deg performs better than STALS.

### 5.6. Parameter K

**Figure 3:**
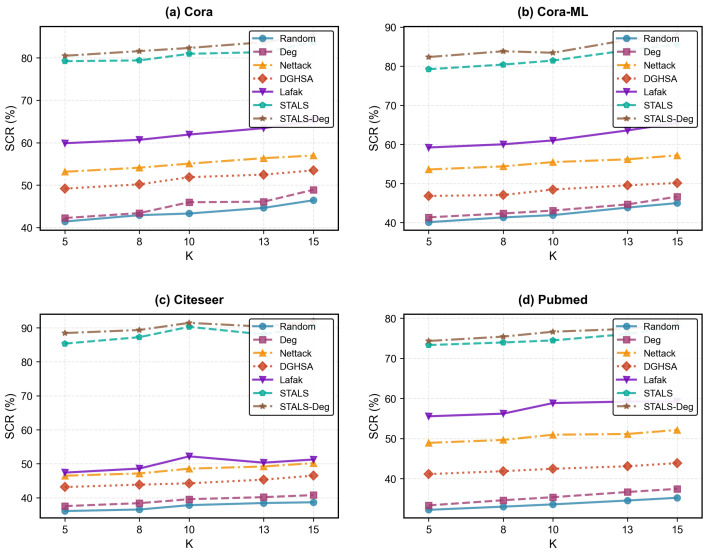
Effect of different parameter K on SCR. The results for each dataset represent the average of all source labels.

Figure 3 reports the effect of the K parameter in HGNNs-KNN on attack performance, with K values selected as {5, 8, 10, 13, 15}. We observe that attack performance increases as the K parameter grows. Regardless of dataset type or K value, the effectiveness of attack strategies consistently follows a fixed priority order from strongest to weakest: STALS-Deg > STALS > Lafak > Nettack > DGHSA > Deg > Random. When K = {5, 10, 15}, the attack classification rates for Lafak and STALS are {59.88%, 61.92%, 65.22%} and {79.21%, 80.95%, 83.66%} on Cora, respectively. Intuitively, when K is small (e.g., K = 5), each node’s neighborhood set is highly streamlined, containing only the most central similar nodes. These nodes exhibit strong consistency in features and labels, forming a highly stable local structure. At this point, the model’s decision relies more heavily on core local information, and this information is highly resistant to interference. Attacks must compromise a large number of core nodes to be effective, making them difficult and inefficient. When K increases (e.g., K = 15): the neighbor set of nodes includes more nodes, whose feature and label consistency is weaker, reducing the stability of the local structure and making it easier for attacks to achieve their goals.

### 5.7. Label Budget Factor α

**Figure 4:**
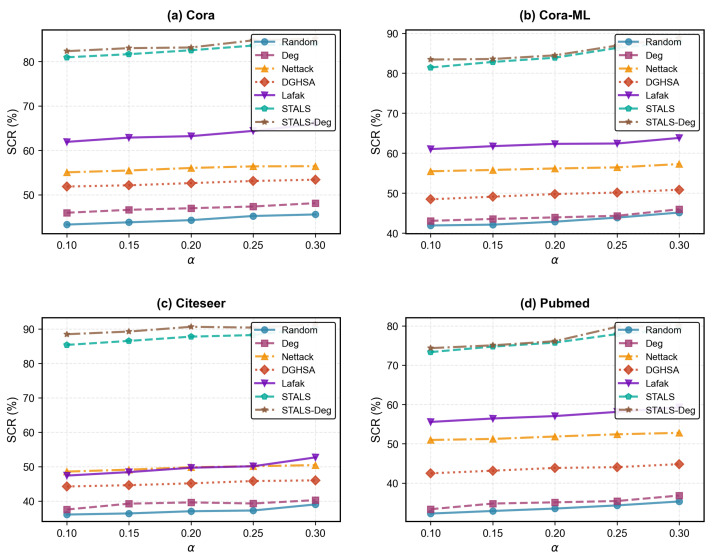
Effect of different parameter α on SCR. The results for each dataset represent the average of all source labels.

This section investigates the performance of attack models under varying label modification budgets. Figure 4 results demonstrate that attack performance increases with larger label modification budgets. For instance, on Cora, when α values are {0.1, 0.2, 0.3}, STALS and STALS-Deg achieve classification accuracies of {80.95%, 82.54%, 84.05%} and {82.33%, 83.16%, 85.15%}, respectively. Notably, our proposed model and its variants perform well under any label change budget, achieving classification accuracy surpassing other comparison models. Taking Citeseer as an example, Lafak, STALS, and STALS-Deg achieve average classification accuracies of 49.65%, 87.62%, and 89.98%, respectively. When the label change budget is high, many nodes’ labels are altered to the target label. During training, HGNNs learn associations between nodes and target labels, enabling the model to misclassify target nodes into the specified label during inference.

### 5.8. Label Budget Factor β

**Figure 5:**
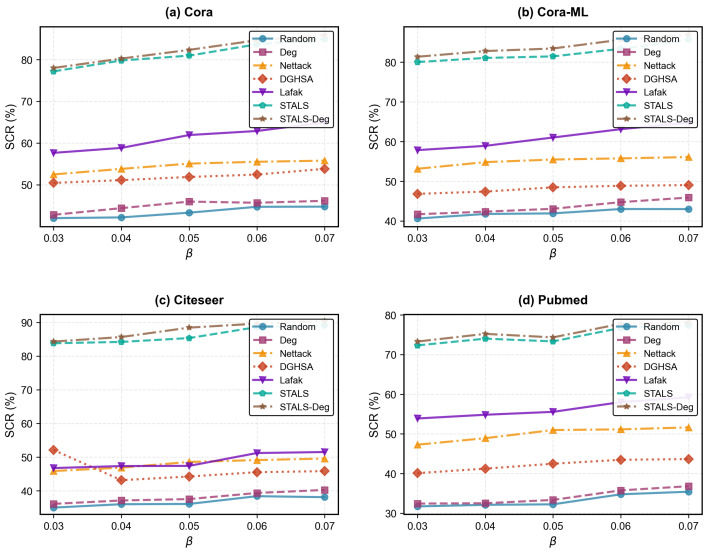
Effect of different parameter β on SCR. The results for each dataset represent the average of all source labels.

This section investigates the impact of structural budgets on attack performance, with results shown in Figure 5. The structural modification budget controls the number of adjustments (add/remove associations) to node–hyperedge associations in the hypergraph. Figure 5 reveals that structural optimization significantly amplifies attack effectiveness, with performance increasing as the budget grows. For example, in Cora, STALS achieves a 77.18% SCR at a budget factor of 0.03, rising to 84.92% at 0.07. STALS-Deg increased from 78.00% to 85.91%. Similar results were observed across other datasets. These findings indicate that label poisoning alone disrupts the alignment between poisoned nodes and original hyperedge labels, hindering error propagation. In contrast, structural modification identifies optimal hyperedge adjustment directions via gradient analysis, enhancing the alignment between poisoned nodes and hyperedges with target labels, thereby accelerating the propagation of error labels at higher levels.

### 5.9. Transferability

In this section, we set the victim model to HGNN-ε to validate the effectiveness of our approach, with results shown in Table 4. Table 4 reports that our proposed method achieves optimal performance on HGNN-ε as well. For instance, when ε = 0.5, the classification accuracies of Deg, Lafak, STALS, and STALS-Deg on Cora-ML are 44.52%, 62.16%, 83.15%, and 85.33%, respectively. Furthermore, larger ε values increase similarity among nodes within hyperedges. Table 4 demonstrates an inverse relationship between overall classification accuracy and ε—higher ε values yield poorer attack performance. For ε = {0.4, 0.5, 0.6}, STALS-Deg achieves classification accuracies of {79.12%, 76.10%, 76.99%} on PubMed. As ε increases, the number of nodes connected by each hyperedge grows, forming more and longer information propagation paths within the hypergraph. Disturbances to a small number of nodes spread rapidly through hyperedges to the local neighborhood of target nodes, disrupting the model’s learning of global features and naturally enhancing attack performance.

## 6. Conclusions

This paper addresses the research gap in strong target attacks against HGNNs and the inability of existing attacks to precisely control target labels. We propose a parameter poisoning-based strong target label attack framework, named STALS. STALS achieves the first-ever directed misclassification of source label nodes into specified target labels, providing critical technical support and risk references for adversarial security research on HGNNs. STALS comprises optimal target label selection and structural adjustment training modules. Specifically, it identifies optimal target labels by integrating feature similarity and hypergraph structural adaptability. Under label alteration budget constraints, it performs label poisoning on source label nodes. Combined with a gradient-guided greedy hyperedge reconstruction strategy, STALS iteratively optimizes the association between poisoned nodes and hyperedges within structural modification budgets to maximize mislabeled information propagation efficiency. Across four benchmark datasets, STALS and its variants significantly outperformed baseline methods.

It is worth noting that the current STALS framework assumes a global-knowledge scenario with high attacker access privileges, where both node labels and hypergraph structures are manipulable. From a security perspective, the performance of STALS under limited-knowledge scenarios (e.g., attackers are restricted from modifying labels and can only rely on structural perturbations) is an important practical extension direction. Preliminary theoretical analysis indicates that the attack performance will decline significantly in such scenarios: the lack of label poisoning removes the explicit guidance for erroneous information propagation, and structural perturbations alone can only disrupt the original feature aggregation rules of HGNNs, making it difficult to achieve precise directed misclassification. This decline will be more obvious for large-scale sparse datasets such as PubMed due to fewer node–hyperedge association paths. In addition, regarding the computational efficiency of the structural adaptability score, its time complexity is linearly related to the number of hyperedges and classes. For large datasets like PubMed, this calculation can be completed in milliseconds on general hardware, verifying the practical applicability of the framework.

Future research may explore the following directions: (1) Extend strong target attacks from semi-supervised node classification to other tasks such as hypergraph clustering and link prediction, investigating adaptation strategies for different tasks. (2) Explore the strong target attack strategy under limited-knowledge/black-box scenarios: Focus on the attack scenario where label modification is prohibited, design more targeted structural perturbation methods to compensate for performance degradation, and improve the practical security research of HGNNs in real-world low-access environments.

## Figures and Tables

**Table 1:** Common parameters and their meanings.

Symbol	Description
α	Label modification budget factor
β	Structural modification budget factor
*T*	Attack iterations
*K*	Parameter in HGNN-KNN
ε	Parameter in HGNN-ε
*L*	Number of layers in HGNNs
η	Learning rate of HGNNs
λ	Decay factor of HGNNs
*h*	Feature dimension of HGNNs
$\Delta_{L}$	Label modification budget
$\Delta_{S}$	Structural modification budget
*C*	Total number of classes
*N*	Total number of nodes
*M*	Total number of hyperedges
*d*	Node feature dimension
SCR	Success Classification Rate
*H*	Clean hypergraph
$H^{\prime }$	Perturbed hypergraph
*V*	Node set of the hypergraph
*E*	Hyperedge set of the hypergraph
*X*	Node feature matrix
$y^{*}$	Predefined target label
$V_{s}$	Set of source label training nodes
$V_{poison}$	Set of poisoned nodes
$\mu_{c}$	Label feature mean
$\rho_{c}$	Hypergraph density for label *c*
$L_{attack}$	Attack loss

**Table 2:** Information on the four datasets. Note: Targeted Class denotes the unique optimal target label selected for each dataset via the feature similarity and hypergraph structural adaptability metrics, which is the predefined target label for strong target attack of the corresponding dataset. Only one optimal target label is calculated for each dataset in the experiment.

Dataset	Nodes	Features	Classes	Targeted Class
Cora	2708	1433	7	2
Cora-ML	2995	2879	7	4
Citeseer	3327	3703	6	4
PubMed	19,717	500	3	2

**Table 3:** Success classification rate (%) for several attack types. Victim HGNNs is HGNNs-KNN. Each result is the average of ten runs, with the optimal value in bold. Note: The symbol “-” indicates that the corresponding source label is consistent with the optimal target label (Targeted Class in Table 2) of the dataset. According to the constraint of Equation (14), there is no valid candidate target label for this source label, so the strong target attack experiment is not conducted for it.

Datesets	Source Label	Random	Deg	Nettack	DGHSA	Lafak	STALS	STALS-Deg	Datesets	Source Label	Random	Deg	Nettack	DGHSA	Lafak	STALS	STALS-Deg
Cora	1	40.64	45.31	55.45	50.15	60.61	78.26	**79.61**	Cora-ML	1	43.21	44.31	57.11	51.78	62.51	79.20	**81.23 **
	2	-	-	-	-	-	-	-		2	44.30	45.52	56.20	49.64	60.18	81.32	**82.31 **
	3	43.61	46.31	54.14	52.84	63.02	86.52	**88.15 **		3	40.64	42.10	54.33	46.27	59.48	83.68	**85.31 **
	4	45.48	47.61	56.03	51.96	62.51	84.60	**85.15 **		4	-	-	-	-	-	-	-
	5	43.61	45.51	51.56	47.83	61.56	77.98	**79.31 **		5	39.88	41.05	54.50	46.71	62.26	82.28	**84.45**
	6	42.98	44.78	56.11	47.43	62.15	78.89	**80.30 **		6	41.54	42.64	50.49	47.12	60.20	81.45	**83.61 **
	7	43.56	46.31	57.17	51.03	61.64	79.45	**81.64 **		7	42.70	43.87	56.26	49.33	61.31	80.69	**83.66 **
Citeseer	1	37.91	39.18	47.37	43.82	51.61	87.53	**89.61 **	PubMed	1	31.88	33.61	49.84	41.19	58.56	75.31	**77.54 **
	2	37.61	38.12	46.52	41.96	50.30	89.75	**91.61 **		2	-	-	-	-	-	-	-
	3	39.61	40.61	51.10	46.25	53.21	88.85	**89.69 **		3	35.31	37.12	52.11	43.78	59.15	73.64	**75.75 **
	4	-	-	-	-	-	-	-									
	5	38.31	41.53	50.66	46.12	54.02	93.43	**93.79 **									
	6	35.64	38.61	47.14	43.07	51.78	91.85	**92.34 **									

**Table 4:** Comparison of model performance across different datasets at various ε-values. Each result is the average of ten runs, with the optimal value in bold.

Models	ε=0.4				ε=0.5				ε=0.6			
	Cora	Cora-ML	Citeseer	PubMed	Cora	Cora-ML	Citeseer	PubMed	Cora	Cora-ML	Citeseer	PubMed
Random	44.24	44.33	38.15	35.23	43.31	43.73	37.49	33.51	42.87	42.59	36.82	32.54
Deg	45.38	45.59	41.99	36.56	44.99	44.52	40.15	35.10	44.61	43.23	39.87	34.68
Nettack	55.14	57.48	49.19	54.21	54.89	56.78	48.48	53.89	53.01	55.78	46.17	52.33
DGHSA	49.54	47.88	46.82	49.16	48.78	45.70	45.57	47.43	46.74	45.01	44.43	46.01
Lafak	62.39	62.98	51.35	59.63	61.21	62.16	50.38	57.05	60.13	61.55	49.66	55.54
STALS	81.78	85.93	90.43	77.64	80.54	83.15	89.02	75.74	80.09	82.02	88.65	74.04
STALS-Deg	**82.50 **	**87.45**	**92.37 **	**79.12 **	**82.16 **	**85.33 **	**90.23 **	**76.10 **	**80.58 **	**84.68 **	**89.24 **	**76.99 **

## Data Availability

No new data were created or analyzed in this study. Data sharing is not applicable to this article.
